# Remission-Stage Ovarian Cancer Cell Vaccine with Cowpea Mosaic Virus Adjuvant Prevents Tumor Growth

**DOI:** 10.3390/cancers13040627

**Published:** 2021-02-05

**Authors:** Courtney T. Stump, Gregory Ho, Chenkai Mao, Frank A. Veliz, Veronique Beiss, Jennifer Fields, Nicole F. Steinmetz, Steven Fiering

**Affiliations:** 1 Department of Biological Sciences, Dartmouth College, Hanover, NH 03755, USA; courtney.t.stump.20@dartmouth.edu; 2 Department of Microbiology and Immunology, Geisel School of Medicine, Dartmouth College, Lebanon, NH 03756, USA; gregory.w.ho.gr@dartmouth.edu (G.H.); chenkai.mao.gr@dartmouth.edu (C.M.); 3 School of Medicine, Case Western Reserve University, Cleveland, OH 44106, USA; frank.veliz@case.edu; 4 Department of Nanoengineering, University of California, San Diego, La Jolla, CA 92093, USA; vbeiss@ucsd.edu (V.B.); nsteinmetz@ucsd.edu (N.F.S.); 5 Norris Cotton Cancer Center, Dartmouth Hitchcock Medical Center, Lebanon, NH 03756, USA; jennifer.l.fields@dartmouth.edu; 6 Moores Cancer Center, University of California, San Diego, La Jolla, CA 92037, USA; 7 Department of Radiology, University of California, San Diego, La Jolla, CA 92093, USA; 8 Department of Bioengineering, University of California, San Diego, La Jolla, CA 92093, USA; 9 Center for Nano-ImmunoEngineering, University of California, San Diego, La Jolla, CA 92093, USA; 10 Institute for Materials Discovery and Design, University of California, San Diego, La Jolla, CA 92093, USA

**Keywords:** ovarian cancer, immunotherapy, vaccine, CPMV, T cell-dependent, adjuvant, nanoparticle, cowpea mosaic virus

## Abstract

**Simple Summary:**

Ovarian cancer survival rates are poor, with most deaths occurring from cancer recurrence following initial remission. Accordingly, there is a significant need for treatments that prevent relapse. Here, using a therapeutic vaccine against a mouse model of ovarian cancer, we evaluate a personalized vaccine that could be delivered to patients during their remission period. We show that mice that receive a combination of cowpea mosaic virus nanoparticles (CPMV) and irradiated tumor cells overwhelmingly reject tumor challenges in a T cell-dependent manner. Accordingly, we extend the demonstrated potential of CPMV as a vaccine adjuvant. We provide initial evidence that vaccines delivered during periods of clinical remission, using previously resected tumor tissue and an immune adjuvant, may comprise a feasible strategy of ovarian cancer treatment.

**Abstract:**

Ovarian cancer is the deadliest gynecological malignancy. Though most patients enter remission following initial interventions, relapse is common and often fatal. Accordingly, there is a substantial need for ovarian cancer therapies that prevent relapse. Following remission generated by surgical debulking and chemotherapy, but prior to relapse, resected and inactivated tumor tissue could be used as a personalized vaccine antigen source. The patient’s own tumor contains relevant antigens and, when combined with the appropriate adjuvant, could generate systemic antitumor immunity to prevent relapse. Here, we model this process in mice to investigate the optimal tumor preparation and vaccine adjuvant. Cowpea mosaic virus (CPMV) has shown remarkable efficacy as an immunostimulatory cancer therapy in ovarian cancer mouse models, so we use CPMV as an adjuvant in a prophylactic vaccine against a murine ovarian cancer model. Compared to its codelivery with tumor antigens prepared in three other ways, we show that CPMV co-delivered with irradiated ovarian cancer cells constitutes an effective prophylactic vaccine against a syngeneic model of ovarian cancer in C57BL/6J mice. Following two vaccinations, 72% of vaccinated mice reject tumor challenges, and all those mice survived subsequent rechallenges, demonstrating immunologic memory formation. This study supports remission-stage vaccines using irradiated patient tumor tissue as a promising option for treating ovarian cancer, and validates CPMV as an antitumor vaccine adjuvant for that purpose.

## 1. Introduction

A serous ovarian carcinoma diagnosis carries a dismal prognosis. Due to the cancer’s nonspecific clinical symptoms, the majority of patients are diagnosed with stage III or stage IV disease, among which the five-year survival rates are 42% and 26%, respectively [1]. The current standard of care includes surgical debulking, during which large quantities of tumor are removed from the peritoneal cavity. Surgery is generally followed by carboplatin and paclitaxel chemotherapy and, while most patients enter remission, many later relapse with chemo-resistant tumors [2]. Following relapse, most patients succumb to their disease. Accordingly, there is a significant need for therapies that could be applied during remission to prevent relapse when the tumor burden is very low.

Cancer immunotherapies have revolutionized clinical oncology, particularly in the treatment of certain cancers, such as melanoma. However, traditional immunotherapies, such as immune checkpoint blockades, have proven largely unsuccessful in treating ovarian cancer [3,4,5,6]. This is likely due to the fact that the ovarian tumor microenvironment is strongly immunosuppressive [7]. While immune checkpoint blockade therapies rely upon revitalizing an existing T cell response, the intense immunosuppression provided by the abundance of M2-type tumor-associated macrophages (TAMs), regulatory T cells (Tregs), and myeloid-derived suppressor cells (MDSCs) in the ovarian cancer microenvironment generally prevents a robust and effective T cell response [8,9,10,11,12,13].

Immune checkpoint blockade therapies have not successfully treated ovarian cancer, and the field is actively investigating other ovarian cancer immunotherapies, including vaccines [14]. An autologous dendritic cell vaccine against mucin 1 (MUC-1), a tumor-associated antigen (TAA), had promising results in phase II clinical trials [15]. Another promising approach relevant to this study used hypochlorous acid-oxidized ovarian whole tumor lysates to treat dendritic cells, ultimately inducing an anti-tumor CD8+ T cell response and extending survival outcomes [16]. Peptide vaccines, such as those directed against another TAA, NY-ESO-1, have shown promise [17,18,19]. Peptide vaccines, or antibodies targeting another TAA relevant to ovarian cancer, sperm surface protein 17 (Sp17), have been studied in mice and humans [20,21,22]. Further, PANVAC is a therapeutic poxviral vaccine containing the genes for the tumor-associated antigens MUC-1 and carcinoembryonic antigen (CEA), as well as immunostimulatory genes CD80, intracellular adhesion molecule-1 (ICAM1), and leukocyte function-associated antigen-3 (LFA3) [23]. It was recently shown that the prophylactic injection of freeze–thawed lysates of a murine ovarian cancer stem-like cell expressing high levels of ROR-1 increased mouse survival [24]. Another study indicated that the prophylactic injection of a TAA, Sp17, and CpG oligodeoxynucleotide, a toll-like receptor (TLR) 9 agonist, dramatically extended survival in mice [25]. Although moderately efficacious, all of these vaccines are therapeutic and designed to treat active disease. This study focused on vaccines designed to be delivered during remission, when disease is clinically undetectable, an approach that we call “remission-stage vaccines”, and we model with prophylactic vaccines.

Cowpea mosaic virus (CPMV) is a 30 nm icosahedral nanoparticle, which contains a bipartite ssRNA genome within a protein capsid and does not have an envelope [26]. Studies by our labs and others have shown that CPMV nanoparticles are immunostimulatory and are recognized by TLRs 7/8 [27,28]. Compared to tobacco mosaic virus, the in-situ delivery of CPMV in a mouse model of melanoma significantly increased survival [29]. The in-situ delivery of CPMV in a murine model of lung melanoma conferred a strong survival benefit that depended upon neutrophils, adaptive immune cells, IL-12, and IFNγ [28]. CPMV has also proven efficacious when delivered as an in-situ vaccine in mouse models of intracranial glioma and breast cancer [30,31]. Furthermore, either standard or slow-release versions of CPMV delivered intraperitoneally significantly delayed the growth of established murine ovarian tumors in the highly aggressive ID8/VEGFA/defb29 model [28,32]. In this same model, the presence of pre-existing anti-CPMV antibodies in mice pre-exposed to CPMV significantly increased survival [33]. CPMV increased the levels of IL-6, TNFα, IFNγ, and GM-CSF produced by non-adherent cells from the peritoneal cavities of treated mice ex vivo, while decreasing levels of TGFβ and IL-10. It also increased the levels of tumor-infiltrating neutrophils and activated dendritic cells [34]. Furthermore, CPMV delivered in combination with radiation therapy induced dramatic tumor regression in the same murine ovarian cancer model [35].

Based on CPMV’s efficacy as a therapy in early-stage established murine ovarian cancer, and its immunostimulatory properties, we use CPMV as an adjuvant to model treatment during patients’ periods of remission. We propose to use the tumor as a remission-stage vaccine antigen source. By using the tumor as the antigen source, the vaccine is fully personalized. Our approach is particularly attractive in the ovarian cancer context because the vast majority of serous ovarian cancer patients undergo surgical debulking, which generates hundreds of grams or multiple kilograms of patient tumor, which is currently discarded. We suggest that this tumor could be retained, disaggregated, inactivated to ensure cells cannot divide, and frozen for future use as the antigen source in a remission-stage vaccine. The inactivated tumor tissue will contain many of the tumor-associated antigens or neoantigens that would be carried by tumors during disease relapse. When combined with adjuvant, the treated tumor tissue could be administered to patients with the goal of preventing fatal relapse. In the murine context, we modeled this as a prophylactic vaccine. With that in mind, we performed these studies that address two central issues: (1) how best to inactivate the tumor so it cannot grow in the patient but retains optimal immunogenicity, and (2) how to pair that inactivated tumor tissue with the ideal vaccine adjuvant. 

In this investigation, murine tumor cells were prepared in four different ways (irradiation, freeze–thawed lysates, heat-shocked lysates, and HOCl-oxidized lysates) to enhance the immunogenicity of the vaccine’s antigen. [16,36,37,38,39,40,41,42,43]. We used CPMV as an adjuvant and compared it to monophosphoryl lipid A (MPLA), a bacterial cell wall component and potent TLR 4 agonist that is an FDA-approved vaccine adjuvant [44,45]. We also compared CPMV to DMXAA, a murine STING agonist and antivascular agent [46]. Following the administration of two vaccines, the mice were challenged with live murine ovarian cancer cells, with survival used as a measure of vaccine efficacy.

Reported here are the results of investigating multiple tumor cell treatments and adjuvants to identify the optimal tumor vaccine. We observed that mice treated with the combination of CPMV and irradiated cells generated a robust T cell-dependent response, which induced superior survival against live tumor cell challenge and subsequent rechallenge, indicating the formation of immune memory. Here, we show that the codelivery of CPMV nanoparticles and lethally irradiated ovarian cancer cells could form the basis of an effective remission-stage ovarian cancer vaccine. 

## 2. Results

### 2.1. CPMV Is an Effective Adjuvant When Combined with Irradiated Syngeneic Ovarian Cancer Cells

MPLA is an FDA-approved vaccine adjuvant that stimulates TLR 4 on antigen-presenting cells (APCs) [44,45]. Furthermore, because MPLA is widely used and easily obtained, it serves as a useful point of reference against which CPMV, the novel adjuvant, can be compared [47,48]. We compared the efficacies of these adjuvants in the ID8/VEGFA/defb29 ovarian cancer cell line, which is aggressive and closely mirrors human serous ovarian carcinoma; it is poorly immunogenic, metastasizes throughout the peritoneal cavity, and causes syngeneic C57BL/6J mice to rapidly develop acute ascites, making it an ideal model of serous ovarian cancer in humans [49].

**Figure 1:**
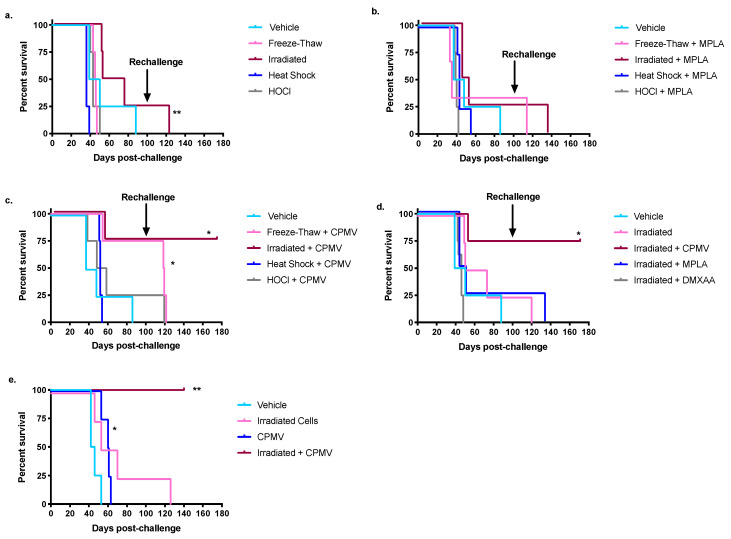
The combination of cowpea mosaic virus nanoparticles (CPMV) and irradiated cells significantly extends survival in a mouse model of ovarian cancer. ID8/VEGFA/defb29 cells were inactivated for intraperitoneal (IP) vaccine injection in one of four different ways: irradiation, freeze–thaw, heat shock, or HOCl oxidation (see methods for cell preparation). Following two vaccinations seven days apart, mice were challenged with live tumor cells and survival tracked. (**a**) Irradiated *p* = 0.007 compared to freeze–thaw, *n* = 4 in all groups; (**b**) cells co-delivered IP with 100 µg MPLA. Irradiated *p* = 0.35 compared to freeze–thaw and *p* = 0.59 compared to vehicle, *n* = 4 in all groups except freeze–thaw + MPLA where *n* = 3; (**c**) inactivated cells were co-delivered IP with 100 µg CPMV, *n* = 4 in all groups, freeze–thaw *p* = 0.03, irradiated *p* = 0.03 compared to vehicle; (**d**) mice received irradiated ID8/VEGFA/defb29 cells co-delivered IP with PBS, 100 µg CPMV, 100 µg MPLA, or 250 µg DMXAA. *n* = 4 in all groups except irradiated + DMXAA where *n* = 8. Irradiated + CPMV *p* = 0.03 or less when compared to any other group; (**e**) *n* = 4 in all groups. Irradiated + CPMV *p* = 0.007 or less compared to any other group. (**a**–**e**) When twice the average length of the survival of vehicle-treated mice had passed, surviving mice were rechallenged with 5 × 10^6^ cells, as denoted by the arrows. *p* values compare survival curves with a log-rank (Mantel–Cox) test. All *p* values are compared to vehicle-treated controls unless otherwise noted ** 0.001 < *p* < 0.01; * 0.01 < *p* < 0.05.

To compare the efficacy of CPMV and MPLA as adjuvants against the ID8/VEGFA/defb29 cell line, they were co-administered with a variety of different antigen preparations. Each immune adjuvant was co-delivered intraperitoneally (IP) with irradiated tumor cells, freeze–thawed tumor lysates, heat-shocked tumor cell lysates, or HOCl-oxidized tumor cell lysates. All antigen preparations were selected because they dependably kill the tumor cells and have previously been found to increase tumor cell immunogenicity [16,36,37,38,39,40,41,42,43]. Mice were given two identical vaccines one week apart, followed by a live tumor cell challenge one week after the second vaccine. We followed the survival of mice given different antigen and adjuvant combinations after their live tumor cell challenge (Figure 1).

Without adjuvant, there was a modest survival advantage provided by the irradiated tumor cells, but none of the other cell preparations yielded a statistically significant survival benefit (Figure 1a). This suggested that, of the preparations tested, radiation was the best option, and combination with adjuvant would strengthen its efficacy. MPLA is a weakly effective adjuvant against the ID8/VEGFA/defb29 murine ovarian cancer cell line when combined with irradiated cells (Figure 1b). Indeed, none of the tumor antigen preparations in combination with MPLA conferred a significant survival advantage beyond the survival of mice given the same antigen preparations without adjuvant. MPLA was not an effective adjuvant in combination with irradiated tumor cells or freeze–thawed lysates, as it did not provide a survival benefit when compared to vehicle-treated mice (Figure 1b) (*p* = 0.59 and *p* = 0.57, respectively). Mice treated with HOCl-oxidized cells and MPLA lived roughly as long as mice treated with HOCl-oxidized cells alone, showing that MPLA is not an effective adjuvant when combined with HOCl-oxidized cells (Figure 1a,b) (*p* = 0.82). Groups treated with heat-shocked lysates in combination with MPLA showed no significant difference between their survival and that of the vehicle-treated mice (*p* = 0.81) (Figure 1b). Because the vaccines that included MPLA as an adjuvant were ineffective, we performed experiments changing the MPLA dose, the amount of antigen included in the vaccine, and the route of injection, but all formulations remained ineffective (Figure S1). We also investigated the combination of irradiated cells and DMXAA, a murine STING agonist, but it, too, did not extend mouse survival (*p* = 0.28) (Figure 1d). 

Like MPLA, CPMV lacked consistent efficacy when combined with heat-shocked tumor lysates or HOCl-oxidized tumor lysates (Figure 1c) (*p* = 0.80 and *p* = 0.53, respectively). The combination of CPMV and freeze–thawed lysates initially appeared effective, since 75% of the mice survived to rechallenge (Figure 1c) (*p* = 0.02). However, despite the significant survival benefit, the mice succumbed to their rechallenge within the expected forty-day timeline, suggesting that they did not mount a protective memory response (Figure 1c).

When combined with irradiated cells, CPMV significantly extended mouse survival and outperformed both MPLA and DMXAA as an adjuvant (Figure 1c,d) (*p* = 0.03 compared to MPLA and *p* = 0.003 compared to DMXAA). Overall, then, compared to MPLA and DMXAA, CPMV provided a far superior survival benefit to mice in the ID8/VEGFA/defb29 model, particularly in combination with irradiated cells. 

In the ID8/VEGFA/defb29 ovarian cancer model the best vaccine combined CPMV with irradiated tumor cells. Compared to the other vaccines tested, the co-administration of CPMV and irradiated cells extended survival the longest, was the only vaccine that enabled total tumor rejection in any mice, and provided mice with the ability to withstand rechallenge, justifying further characterization.

To confirm that both antigen and adjuvant, or in this case irradiated cells and CPMV, were necessary for vaccine efficacy, the survival of mice vaccinated with both irradiated cells and CPMV was compared to mice vaccinated with irradiated cells alone or CPMV alone. Both CPMV and irradiated cells were required to confer survival benefit against the ID8/VEGFA/defb29 cell line (Figure 1e). At 140 days post-challenge, which is over three times as long as it generally takes for the vehicle-treated mice to reach the endpoint, all mice vaccinated with both irradiated cells and CPMV remained alive and tumor-free (*p* = 0.006). In contrast, by day 65, all mice treated with CPMV alone had reached the endpoint criteria. While we did observe that CPMV treatment alone provided a statistically significant survival benefit, the benefit was not comparable to that provided by the complete vaccine (Figure 1e) (*p* = 0.02). Similarly, all mice treated with irradiated cells alone succumbed to their cancer. Compared to CPMV alone and to irradiated cells alone, the combination vaccine significantly increased survival (*p* = 0.007 for each), indicating that both irradiated cells and CPMV are necessary for full vaccine efficacy. 

**Figure 2:**
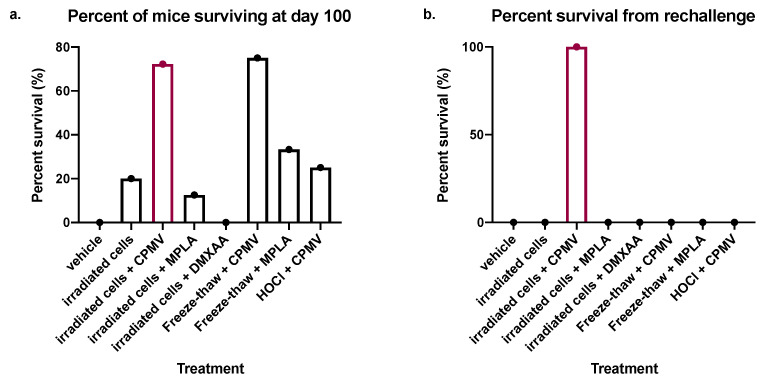
The prophylactic codelivery of irradiated cells and CPMV provides robust, long-term protection from ID8/VEGFA/defb29 tumor challenge and rechallenge. (**a**) Combination data from all survival experiments. Mice received treated ID8/VEGFA/defb29 cells, co-delivered IP with or without adjuvant. The panel shows the percent of mice (out of the total number that received that treatment in all experiments) that survived to day 100 to be rechallenged. (**b**) The panel shows the percent of mice that survived 60 days after being rechallenged. All groups with any mice surviving to rechallenge are included, as are all groups that included irradiated cells and all vehicle-treated mice. (**a**,**b**) Vehicle-treated *n* = 24, irradiated cells *n* = 20, irradiated cells + CPMV *n* = 20, irradiated cells + MPLA *n* = 8, irradiated cells + DMXAA *n* = 8, freeze–thaw + CPMV *n* = 4, freeze–thaw + MPLA *n* = 3, HOCl + CPMV *n* = 4.

We selected survival for 100 days after a challenge with ID8/VEGFA/defb29 tumor cells as the indication of rejection of the challenge. While vehicle-treated control mice reached endpoint criteria around 40 days post-challenge, over 40% of vaccine-treated mice in this study survived for 100 days. CPMV provided extremely dependable protection against rechallenge when combined with either irradiated cells or freeze–thawed cells, with 75–100% of mice given those vaccines surviving for 100 days or more with no signs of ascites development (Figure 1c–e). However, other treatments also sporadically enabled the survival of challenged mice for 100 days at frequencies between 25 and 33% in some experiments, namely, irradiated cells only (Figure 1a), freeze–thaw or irradiated + MPLA (Figure 1b,d), HOCl + CPMV (Figure 1c), and irradiated cells only (Figure 1a,d,e). In Figure 2a, we show the total percent survival of all mice at 100 days. These composite data clearly established the combination of CPMV and irradiated cells, or CPMV and freeze–thawed cells, as the best vaccine to mediate resistance to the primary challenge, and these treatments were roughly equal in protecting mice from primary tumor challenge.

While protection from the primary tumor challenge is an important assessment of vaccine efficacy, the establishment of protective immune memory is also very important. Accordingly, mice that survived the primary challenge for 100 days with no sign of ascites development were rechallenged to assess their ability to reject tumors months after vaccination, providing an indication of immune memory. The rechallenge data for each experiment and subsequent survival are shown in Figure 1a–d. We selected survival for at least 60 days following rechallenge as an indication of established, protective immune memory. Only mice treated with the combination of CPMV and irradiated cells survived 60 days following rechallenge (Figure 1 and Figure 2b). This study of longer-term protection clearly established the combination of CPMV and irradiated cells as superior to any other vaccine, including the combination of freeze–thawed cells and CPMV. Additionally, two mice vaccinated with CPMV and irradiated cells that survived 100 days after rechallenge (200 days after primary challenge) were rechallenged a second time at that 200 day mark, after which they survived to 300 days, when they were rechallenged for the third time. Both animals survived until 450 days after the primary challenge and had no ascites when the experiment was concluded (data not shown). Overall, these data show the unique ability of the vaccine combining CPMV and irradiated cells to establish long-term, protective memory against a mouse model of ovarian cancer.

### 2.2. The Survival Benefit Provided by the Combination of CPMV and Irradiated Cells Is T Cell-Dependent

**Figure 3:**
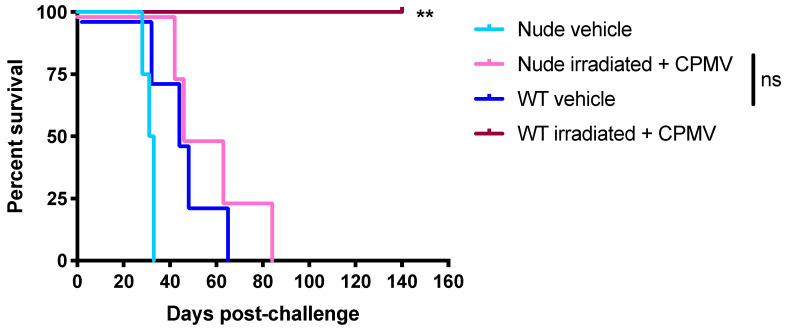
The survival benefit provided by the co-delivery of CPMV and irradiated cells is T cell-dependent. Irradiated ID8/VEGFA/defb29 cells were co-delivered IP with 100 µg CPMV to C57BL/6J (WT) or NU/J (nude) mice. Following two vaccinations seven days apart, mice were challenged with 5 × 10^6^ live tumor cells. *n* = 4 in all groups. Compared to WT vehicle: WT irradiated + CPMV *p* = 0.007, nude irradiated + CPMV *p* = 0.29, and nude vehicle *p* = 0.10. Compared to nude vehicle, nude irradiated + CPMV *p* = 0.009. *p* values compare survival curves with a log-rank (Mantel–Cox) test. All *p* values are compared to vehicle-treated controls unless otherwise noted. ** 0.001 < *p* < 0.01; ns *p* > 0.05.

To begin to understand the immunological mechanisms of the vaccine combining irradiated cells and CPMV, the survival of vaccinated wild-type mice was compared to the survival of vaccinated nude mice that lack T cells. Comparing the vaccine’s efficacy in nude mice to its efficacy in wild-type mice elucidates the importance of T cells to the anti-tumor immune response (Figure 3).

The survival benefit conferred by the vaccine combining irradiated cells is T cell-dependent. Vehicle-treated nude mice succumbed to their tumors very quickly, likely because they lacked even the immune pressure of the anti-tumor T cells in unvaccinated mice (Figure 3). However, the difference in survival between nude unvaccinated mice and wild-type unvaccinated mice was not significant (*p* = 0.10). All vaccinated wild-type mice remained tumor-free at day 140, which was over three times the average survival of vehicle-treated mice, and they experienced a significant survival advantage compared to unvaccinated wild-type mice (*p* = 0.007). The survival curves of the wild-type unvaccinated mice and the vaccinated nude mice closely mirrored one another, and there was no significant difference in the survival between these two groups (*p* = 0.29). While all of the vaccinated wild-type mice rejected their tumors, none of the vaccinated nude mice rejected their tumors, providing clear evidence that the vaccine’s immunological mechanism requires T cells. Perhaps the innate immune activation provided by CPMV allows a protective T cell response to be primed, ultimately causing tumor rejection.

## 3. Discussion

Despite enormous advances in clinical cancer immunotherapies over the last two decades, none have shown clinical efficacy in treating ovarian carcinomas. Though a combination of surgical, chemotherapeutic, and radiological interventions often induces clinical remission, serous ovarian cancer generally returns. Accordingly, there is a significant need for patient-centered immunotherapies that could be delivered during patients’ remission to prevent disease relapse [50]. Immunotherapies, including but not limited to vaccines, have the best opportunity to completely eliminate disease during remission, when low tumor burden leads to relatively weak tumor-mediated immunosuppression. As such, we believe that a vaccine that actually cures disease, rather than one that extends survival, is best delivered during clinical remission before disease relapse. In order for that vaccine to induce a strong immune reaction, it must include an adjuvant. Furthermore, to specifically direct the vaccine against tumor cells, the vaccine must include an antigen source. If immunogenic tumor antigens overlap between the primary tumor removed during surgical debulking and the tumor present at relapse, then the primary tumor serves as a useful antigen source. Because a relatively large amount of tumor tissue is discarded from each patient’s surgery, the tumor tissue removed during surgery serves as a patient-specific and readily available antigen source for a personalized cancer vaccine.

In this investigation, we show that the combination of CPMV and irradiated murine ovarian cancer cells constitutes an effective, T cell-dependent prophylactic vaccination against an aggressive syngeneic mouse model of ovarian cancer. CPMV, an immunostimulatory plant viral nanoparticle that has previously shown promise as a therapeutic agent, was compared to MPLA, a TLR 4 agonist, and DMXAA, a murine STING agonist. CPMV was a consistently more effective adjuvant than either MPLA or DMXAA (Figure 1). To determine the best antigen source for the vaccine, four different tumor preparations were compared—ionizing irradiation, freeze–thawed lysates, heat-shocked lysates, and hypochlorous acid-oxidized lysates—and we observed that irradiated tumor cells were the most effective vaccine antigen. Together, the combination of CPMV and irradiated tumor cells enabled the majority of treated mice to reject the primary tumor challenge, as well as subsequent tumor challenges over a prolonged period. 

Unsurprisingly, both CPMV (the adjuvant) and irradiated cells (the antigen) were necessary for vaccine efficacy, supporting the expected vaccine function (Figure 1e). Most mice vaccinated with irradiated tumor cells and CPMV survived both the original tumor challenge and at least one rechallenge, with 70–75% of vaccinated mice surviving the initial challenge and all mice surviving rechallenge (Figure 1 and Figure 2). The remarkable efficacy of CPMV in extending survival in the highly aggressive ID8/VEGFA/defb29 model is consistent with other studies, which showed that CPMV moderately extended the survival of tumor-bearing mice when delivered therapeutically [32,35,51,52]. Accordingly, our present data suggests a novel application for CPMV as an effective adjuvant in remission-stage vaccines that block ovarian cancer relapse.

This sort of robust response is most often accomplished by CD8+ T cells, and our vaccine was rendered minimally effective in nude mice lacking T cells (Figure 3). Furthermore, since vaccinated mice survived tumor rechallenges delivered 80–100 days after their original tumor challenge, they appear to have robust immunological memory against the ID8/VEGFA/defb29 tumor cell line (Figure 2). While most tumor immunotherapies involve CD8+ T cells, the data we present here are also consistent with a reliance upon CD4+ T cells, either for their own cytokine production or for their ability to help mount a protective B cell response; these possibilities cannot be ruled out [53]. Though more studies are necessary to thoroughly explain the vaccine’s mechanisms, it is clear that the vaccine functions via a T cell-dependent immunological mechanism. These data corroborate earlier findings indicating that a vaccine consisting of CPMV conjugated to NY-ESO-1 induces an antigen-specific CD8+ T cell response [54].

The activation of TLR signaling appears to be involved in CPMV’s efficacy as an adjuvant. CPMV’s mechanism of immune cell activation is not yet fully understood, but CPMV does signal through TLR 7/8 and requires Syk signaling, while MPLA signals through TLR 4; perhaps differences in TLR activation account for the contrasting survival benefits [27]. A number of studies support CPMV’s ability to act as an immune adjuvant for in-situ vaccines [27,28,34,51,52]. Furthermore, CD11b+ monocytes from mouse ascites activated ex vivo with a combination of TLR 4 and TLR 9 agonists moderately extended survival when provided prophylactically, and led to tumor rejection and long-term memory in the therapeutic setting, further underscoring the important role of TLR stimulation in effective ovarian cancer immunotherapies [55]. Accordingly, though more studies are needed to fully elucidate the mechanism of the vaccine we describe here, it seems likely that CPMV activates innate immune cells, allowing the priming of an anti-tumor T cell response.

Vaccines with whole tumor cell antigens have long been an area of interest in ovarian cancer immunotherapies [56,57,58,59]. Of the various antigen preparations that we tested, the ionizing irradiation of tumor cells provided the best survival benefit (particularly in combination with CPMV) (Figure 1a,c). Ionizing radiation has not been used extensively as an antigen preparation technique in the past. However, perhaps most famously, irradiated tumor cells expressing GM-CSF comprise GVAX, an early approach which helped galvanize the field of cancer immunotherapy [60]. Furthermore, irradiation has been found to increase dendritic cell activation and mouse survival compared to freeze–thawed lysates in a dendritic cell vaccine against mouse models of glioma and melanoma [36,37]. Most probably, this is because gamma irradiation increases the expression of tumor antigens or oxidation-associated molecular patterns (specific types of danger-associated molecular patterns), which vigorously activate APCs [60,61]. Furthermore, irradiation increases the expression of the T cell costimulatory molecule CD80 on a variety of tumor cells, which increases tumor cell immunogenicity [62]. These past findings are consistent with our observation that irradiated tumor cells alone (without adjuvant) increased mouse survival (Figure 1a).

Freeze–thawing, heat-shocking, and HOCl-oxidizing tumor cells were generally less effective antigen preparation techniques when combined with CPMV or MPLA. To our knowledge, no studies have examined these techniques in the prophylactic context in ovarian cancer, but the therapeutic literature has suggested that preparing tumor cells in these ways can be advantageous. Freeze–thawed lysates delivered in combination with CPMV initially conferred a survival benefit, but did not establish immunological memory, as mice succumbed to rechallenge (Figure 1c). Freeze–thawed lysates are used regularly and are often used to prepare tumor cell lysates in successful dendritic cell vaccines [43,59,63]. For this reason, we compared various different freeze–thawed lysates to irradiated cells. However, it is possible that freeze–thawed lysates can decrease the ability of dendritic cells to respond to TLR stimulation, which may explain why their combination with CPMV was not as effective as the combination of CPMV and irradiated cells was [64]. Other groups have found that treating ovarian cancer cells with HOCl has improved the ability of dendritic cells to prime anti-tumor T cell responses and extend survival [16,41,42,65,66]. Our results did not corroborate the efficacy of HOCl oxidation as an antigen preparation technique. Because the heat treatment of cells causes an increased expression of immunogenic heat shock proteins, heat-shocked lysates have also been used to induce anti-tumor immune responses. Others have found that heat-shocking tumor cells can intensify the anti-tumor immune response, and they have made effective dendritic cell vaccines against colon cancer with heat-shocked tumor cells [38,39]. Studies have even examined the optimal methods of heat-shocking tumor cells for vaccine preparation, which were used to inform our method of heat-shocking tumor cells [40]. The results from our study do not corroborate previous studies that have found heat-shocked lysates to be effective.

The results we describe here align with previous work regarding the role of T cells in ovarian cancer. Human patients with ovarian cancer are capable of mounting modest anti-tumor CD8+ T cell responses, though clinically these responses do not appear sufficient to protect patients; while it is very difficult to understand the level of response that exists early in disease development, the fact that clinical disease develops suggests that the T cell response is not sufficiently protective [67,68,69,70,71]. Perhaps the strategy modeled here, a combination of robust T cell priming and strategic delivery of the vaccine during times of low tumor burden, would invigorate T cell responses enough to prevent relapse in humans. Furthermore, there is reason to believe that genetically modifying the patient’s own resected tumor tissue as a component of a personalized cancer vaccine would be useful in the clinical setting. When patients’ own autologous whole tumor cells are engineered to co-express GM-CSF and a shRNA against furin, subsequently irradiated, and delivered to ovarian cancer patients during periods of remission, blood samples from treated patients have increased levels of IFNγ, and treated patients experience a significant survival benefit [72].

Mice that received two prophylactic vaccines consisting of irradiated cells and CPMV had a significant survival benefit. We suggest here that, due to the low (clinically undetectable) tumor burden present during remission, prophylactic vaccine delivery provides a model for remission-stage vaccines. However, we acknowledge that prophylactic vaccines do not perfectly model remission-stage vaccines; during clinical remission, the immune system is no longer naïve to cancer antigens, and tumor immune-editing can occur.

Mice treated with the best vaccine combination in this study, irradiated cells and CPMV, experienced a remarkable survival rate compared to other therapies reported in the literature with this model. In many of our trials, 70–75% of vaccinated mice survived the initial challenge and rechallenge, and our total combined cohort indicates that 100% of rechallenged mice survived their rechallenge (Figure 1, Figure 2 and Figure 3). In contrast, other studies examining CPMV in this model cite a more modest survival benefit, with roughly 25% surviving challenge and rechallenge or, more frequently, no mice remaining tumor-free [33,34,51]. 

Immune checkpoint blockade therapies, which are widely used cancer immunotherapies, have at best a 15% overall response rate in ovarian cancer patients [3]. Some of the most promising murine ovarian cancer therapies to date combined various immunotherapies, such as a STING agonist with anti-PD-1 immune checkpoint blockade or GVAX, or FVAX, anti-41BB and anti-PD-1 or PD-L1 [73,74]. Compared to other murine vaccine studies, including dendritic cell vaccines, the vaccine developed in this study confers a much greater survival advantage [16,24,75]. In a study wherein mice were treated with a triple checkpoint blockade therapy, 20% of the mice remained tumor-free [76]. However, another group that engineered CAR T cells with the NKG2D receptor observed excellent mouse survival rates in ovarian cancer, and is moving toward clinical trials [77,78]. One of the most promising prospective ovarian cancer vaccines targets Sp17 and utilizes CpG, a TLR 9 agonist, as an adjuvant [25]. While our vaccine provides comparable survival, the predominance of tumor escape in single antigen vaccines has become apparent. Accordingly, the present investigation provides an important, effective direction for the development of multiple antigen-targeted ovarian cancer immunotherapies.

## 4. Materials and Methods 

### 4.1. Animals

Six-week-old female C57BL/6J and athymic nude (NU/J) mice were purchased from The Jackson Laboratory (Bar Harbor, ME, USA). All mice were housed in the Norris Cotton Cancer Center vivarium in accordance with Institutional Animal Care and Use Committee guidelines.

### 4.2. Tumor Models

The ID8/VEGFA/defb29 murine ovarian serous carcinoma cell line was generated as previously described [49]. Cells were cultured at 37 °C in RPMI complete media (RPMI 1640 (HyClone, Marlborough, MA, USA) supplemented with 10% (*v/v*) fetal bovine serum (Gibco, Waltham, MA, USA), 1 mmol/L sodium pyruvate (Life Technologies, Waltham, MA, USA), 1% (*v/v*) penicillin/streptomycin mixture (Gibco, Waltham, MA, USA), and 2 mmol/L L-glutamine (Gibco, Waltham, MA, USA)). Cells were harvested and washed with RPMI 1640. Eight-week-old mice were challenged with 5 × 10^6^ tumor cells in 400 µL sterile PBS intraperitoneally on day 0 after receiving vaccines on days −14 and −7. After challenge, the mice were weighed regularly to monitor ascites formation. Mice were euthanized with carbon dioxide when they reached the humane endpoint of 33 g of weight, indicating significant ascites formation. Many surviving mice were rechallenged around 100 days after their initial tumor challenge. The mice were never given vaccines following tumor challenge or rechallenge.

### 4.3. Vaccine Antigen Preparation

To prepare freeze–thawed lysates, the cells were washed with PBS and harvested. Cells were transferred to 15 mL conical tubes and resuspended in RPMI complete media. Tubes were submerged in a dry ice–ethanol slurry for 10 min. Cells were then allowed to thaw to room temperature in a room-temperature water bath. The freeze–thaw cycle was repeated five times. The freeze–thaw procedure was adapted from Chiang et al. (2011) and Herr et al. (2000) [43,63]. Cell lysis was confirmed via trypan blue exclusion. Cells were resuspended in sterile PBS at a concentration of 25 × 10^6^ cells per mL. Lysates were injected intraperitoneally on days −14 and −7, with 5 × 10^6^ cells delivered to each mouse simultaneously with adjuvant injection.

To prepare irradiated cells, cells were washed with PBS and harvested. Cells were transferred to conical tubes in RPMI complete media and irradiated with 70 Gray (10 Gy per min for 7 min) ionizing gamma radiation from a cesium source. The preliminary experiments confirmed complete cell death following this radiation procedure. Cells were resuspended in sterile PBS at a concentration of 25 × 10^6^ cells per mL. Cells were placed on ice until just prior to vaccination. Irradiated cells were injected on days −14 and −7, with 5 × 10^6^ cells delivered to each mouse simultaneously with adjuvant injection. 

To prepare heat-shocked lysates, cells were washed with PBS and harvested. Cells were resuspended in RPMI complete media and heat-shocked in a water bath at 43 °C for 30 min. Cells were removed from the water bath and incubated at 37 °C for 1 h. Cells were then subjected to five freeze–thaw cycles, as described above. The heat-shocked lysate procedure was adapted from Ito et al. (2005) [40]. Cells were resuspended in sterile PBS at a concentration of 25 × 10^6^ cells per mL. Lysates were injected intraperitoneally on days −14 and −7, with 5 × 10^6^ cells delivered to each mouse simultaneously with adjuvant injection.

To prepare hypochlorous acid-oxidized lysates, cells were washed with PBS and harvested. Cells were resuspended in 0.06 M HOCl in HBSS and incubated at 37 °C for 30 min. Cells were gently agitated to encourage oxidation, and then returned to the incubator for another 30 min. The HOCl-oxidation procedure was adapted from Chiang et al. (2006) [41]. Cells were centrifuged at 5000 rpm for 5 min and washed twice with PBS. Cells were subjected to five freeze–thaw cycles, as described above, before being resuspended in sterile PBS at a concentration of 25 × 10^6^ cells per mL. Lysates were injected intraperitoneally on days −14 and −7, with 5 × 10^6^ cells delivered to each mouse simultaneously with adjuvant injection. 

Vaccines were entirely prophylactic; mice were never given vaccines after tumor challenge or rechallenge. Two doses were provided due to the preponderance of the literature suggesting that both primary and secondary immune responses are important for prophylactic vaccine efficacy [79,80].

### 4.4. Vaccine Adjuvant Preparation

MPLA (Sigma Aldrich, (St. Louis, MO, USA) was dissolved in 2.5% (*v*/*v*) sterile DMSO in ET-free PBS to a concentration of 0.50 µg MPLA/µL. Each mouse received 200 µL MPLA solution (100 µg MPLA) on day 14 and day 7, simultaneously with antigen injection. DMXAA was dissolved in 2.5% (*v/v*) sterile DMSO in ET-free PBS to a concentration of 1.25 µg MPLA/µL. Each mouse received 200 µL DMXAA solution (250 µg DMXAA) on days −14 and −7, simultaneously with antigen injection [81]. 

CPMV nanoparticles were prepared as previously described and were verified to have < 50 endotoxin units per mg protein [82]. CPMV was diluted in PBS to a concentration of 100 µg per 200 µL PBS. Each mouse received 200 µL CPMV solution (100 µg CPMV) injected intraperitoneally on days −14 and −7, simultaneously with antigen injection. 

### 4.5. Statistical Analysis

All statistical analyses were performed with GraphPad Prism 8 (San Diego, CA, USA). All *p* values reported compare survival curves using the log-rank (Mantel–Cox) test. All experimental curves were compared to the relevant vehicle-treated controls unless otherwise stated. 

## 5. Conclusions

This study shows that remission-stage ovarian cancer vaccines using irradiated tumor cells can effectively and significantly increase survival in a mouse model, suggesting the possibility of a similar potential in human serous ovarian cancer patients. These results suggest that, in other cancers in which patients frequently experience long periods of remission before their cancers recur, the development of inactivated tumor cell vaccines to be delivered during that period of remission could be useful. After testing a variety of tumor cell treatments and established as well as experimental adjuvants, we found that only the combination of CPMV and irradiated cells enabled the vast majority of mice to respond to both the original tumor challenge and the rechallenge. These studies support our proposition of CPMV as a novel tumor vaccine adjuvant. Overall, the combination of irradiated cells and CPMV together provides a protective and T cell-dependent ovarian cancer vaccine against a mouse model of ovarian cancer, and opens doors for future studies in cancer immunotherapy.

## Data Availability

The data presented in this study are available on request from the corresponding author.

## Supplementary Materials

The following are available online at https://www.mdpi.com/2072-6694/13/4/627/s1, Figure S1: MPLA is an ineffective adjuvant against the ID8/VEGFA/defb29 murine ovarian cancer model.
