# Supplementary material for: Remission-Stage Ovarian Cancer Cell Vaccine with Cowpea Mosaic Virus Adjuvant Prevents Tumor Growth
Source: Cancers (Basel). 2021 Feb 5;13(4):627. doi: 10.3390/cancers13040627 (PMC7915664; doi:10.3390/cancers13040627)
Supplement: Supplementary file 1 [file cancers-13-00627-s001.pdf]

Supplementary Material: Remission-Stage Ovarian Cancer Cell Vaccine with Cowpea Mosaic Virus Adjuvant Prevents Tumor Growth

Courtney T. Stump, Gregory Ho, Chenkai Mao, Frank A. Veliz, Veronique Beiss, Jennifer Fields, Nicole F. Steinmetz and Steven Fiering

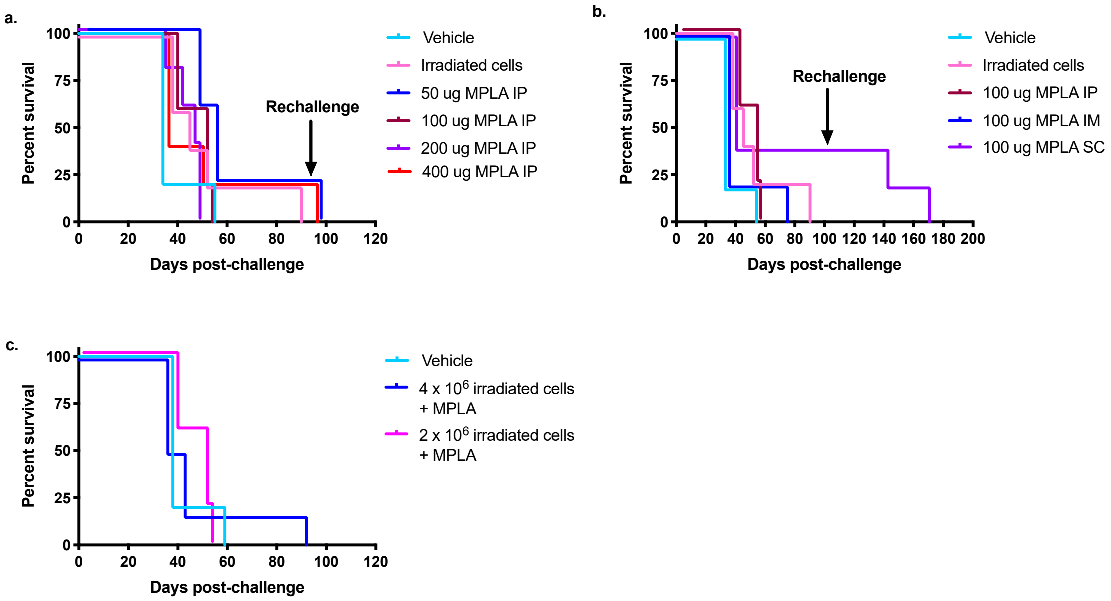

Figure S1. MPLA is an ineffective adjuvant against the ID8/VEGFA/defb29 murine ovarian cancer model. (**a**) Irradiated ID8/VEGFA/defb29 cells were codelivered IP with PBS or various doses of MPLA. n = 5 for all groups; (**b**) Irradiated ID8/VEGFA/defb29 cells were codelivered with PBS or 100 µg MPLA. Antigen and adjuvant were codelivered either intraperitoneally (IP), intramuscularly (IM), or subcutaneously (SC). The vaccines in the vehicle and irradiated cell groups were delivered IP. n = 5 for all groups; (**c**) Irradiated ID8/VEGFA/defb29 cells were codelivered IP with 100 µg MPLA. n = 5 for all groups; (**a**–**c**) All mice received two vaccinations seven days apart. Mice were then challenged with live ID8/VEGFA/defb29 cells delivered IP. Once twice the average length of the survival of vehicle-treated control mice had passed, mice were rechallenged with an equivalent number of live ID8/VEGFA/defb29 cells. No results reached statistical significance ($p < 0.05$).
